# Diversity in anopheline larval habitats and adult composition during the dry and wet seasons in Ouagadougou (Burkina Faso)

**DOI:** 10.1186/1475-2875-9-78

**Published:** 2010-03-19

**Authors:** Florence Fournet, Maud Cussac, Ali Ouari, Pierre-Erwann Meyer, Hyacinthe K Toé, Louis-Clément Gouagna, Roch K Dabiré

**Affiliations:** 1 Institut de Recherche pour le Développement, UMR190, BP 182, Ouagadougou, Burkina Faso; 2 Institut de Recherche en Sciences de la Santé (IRSS), BP 390, Bobo-Dioulasso, Burkina Faso; 3 Laboratoire Espace, Santé et Territoires, Université Paris Ouest la Défense, 92001 Nanterre cedex, France; 4 Institut de Recherche pour le Développement, UR016, BP 545, Bobo-Dioulasso, Burkina Faso

## Abstract

**Background:**

Several cases of malaria are frequently recorded during the dry period in Ouagadougou town (Burkina Faso). This has led to the design of a series of studies focusing on both parasitological and entomological investigations intended to provide relevant health data on the risk of local malaria transmission according to the way of urbanisation.

**Methods:**

A cross-sectional entomological survey was carried out in various districts of Ouagadougou in April and October 2006. Adult malaria vectors were collected using CDC traps and indoor insecticide spraying performed in four houses during four consecutive days/nights. Intensive larval sampling was also done in available water ponds throughout the study sites.

**Results:**

In April, the anopheline breeding sites consisted only of semi-permanent or permanent swamps located mainly in the two peripheral districts. Despite the presence of anopheline larvae in these breeding sites, less than five *Anopheles gambiae *s.l. adults were caught by CDC traps and indoor insecticide spraying. In October, additionally to the permanent breeding sites reported in April, some rainfall swamps were also found positive to anophelines. The number of adults' mosquitoes was higher than that collected in April (2 *vs *159 in October). Out of 115 larvae of *An. gambiae *s.l. analysed by PCR in April, 59.1% (68/115) were identified as *Anopheles arabiensis*, 39.1% (45/115) as *An. gambiae *M while the S form represented less than 2%. Overall 120 larvae and 86 females were identified by PCR in October as *An. gambiae *M form (51%) and *An. arabiensis *(42.2%). The S form represented only 6.8%. The global sporozoite rate recorded was high (6.8%) and did not differ between the districts except in the central district where no positive mosquito was detected.

**Conclusion:**

Although only few adults' mosquitoes were actively caught during the driest month, malaria vectors persisted all year long that increases the risk of urban malaria transmission. The distribution of breeding sites and especially the occurrence of malaria vectors were more abundant in the periphery, which is more like that of a rural settlement. The evolution of malaria prevalence and the factors sustaining the risk of transmission in Ouagadougou as well in many African cities during the dry season are discussed.

## Background

Nowadays, human settlements in many African countries are gradually, but inadequately, experiencing an urbanization process that constitutes a great challenge. More often than not, rapid and unplanned urban growth is the source of many environmental hazards including overcrowding, water shortage and pollution, sub-standard housing, poor solid waste management. Generally, the urban landscape management is expected to reduce the risk of malaria transmission due to the lack of suitable breeding sites for vector development. Indeed, before any insecticide was invented, vector control meant mainly environmental management by drying up the swamps. This is not the case in urban agglomerations in Africa, where the convergent pattern of house dispersion and landscape use is more rural than urban, with precarious peripheral quarters surrounded by gardening and specialized small farming.

One consequence is the multiplication of aquatic habitats induced by the presence of non-formal and disordered hydro-agricultural installations co-occurring permanently with the urbanization and often suitable for vector development. This may increase the risk of vector proliferation in urban areas, where malaria transmission is deemed neglected because theoretically malaria is considered by health planners to be a seasonal and rural problem affecting particularly children. However, the threat of malaria vectors is a major concern for poorly immune populations living in African cities [1]. Indeed, it has been estimated that the 200 million people living in town in Africa, which corresponds to 25% of the African global population, are exposed permanently to malaria infection [1]. Projecting the extension of urbanization process towards 2025, more than 50% of African population will live in towns [2].

In Burkina Faso, available reports indicate that about 40.2% of global morbidity recorded in 2006 in health centres were ascribed to malaria, mainly affecting children aged 0-5 years. In fact, this vulnerable group represents 64.5% of hospitalized patients. On the whole, 45.8% of deaths are caused by malaria [3]. Except for the study by Wang *et al *[4], no study has been explicitly undertaken on malaria epidemiology in Ouagadougou since the end of 1980s. The earlier studies of Rossi *et al *[5] showed that malaria transmission was heterogeneous in the city: it was highest close to the urban dams and also in the peripheral areas and quite nil in the central districts. In order to provide a health database that will be correlated to the urbanization pattern in Ouagadougou town, a global health investigation including parasitological studies were conducted in 2004 [6]. This survey clearly indicated that malaria prevalence averaged 21% for the 0-12 year age group, and varied greatly from one district to another but globally ranged from 31.7% in peripheral quarters to 13% in the central districts. However, complementary entomological data supporting this information is lacking. An increase awareness of the presence of malaria vectors and malaria transmission in Ouagadougou stimulated interest to provide entomological data allowing a better documentation of the database with the intended goal to record the presence of mosquito larval habitats according to urbanization pattern.

The present paper reports the preliminary investigation on the diversity of anopheline breeding sites and species composition in those habitats disseminated throughout the city during the dry season and the end of the rainy season.

## Methods

### Study areas

**Figure 1:**
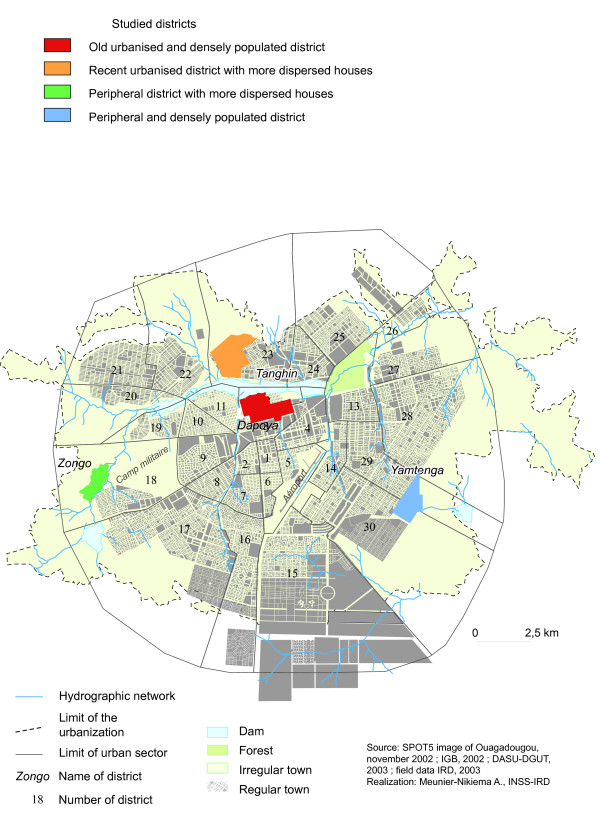
Location of the surveyed districts in Ouagadougou

For the entomological investigations four districts were chosen based on the level of urbanization and the density of housing (Figure 1). The selected sites included i) an old urbanized and densely populated district (Dapoya), ii) a recently urbanized district with more dispersed houses (Tanghin), iii) a peripheral and densely populated quarter (Yamtenga), and iv) a peripheral district with more dispersed houses (Zongo). These parameters were correlated to the dissemination of breeding sites favourable to mosquito development.

**Dapoya **is an old colonial district located in the centre of the town. It is characterized by close-together houses with numerous households. It is surrounded by a large dam collecting polluted domestic water and also rain water. While these water collections dry up progressively during the dry season, they offer fertile areas with some dispersed depressions constituting numerous small water pools. This landscape is much used for vegetable cropping that is intensively developed during the dry period. In addition, numerous wells are also made in the dam bed contributing to collect water for vegetable irrigation. Other domestic water collection channels are disseminated across the quarter. All these natural and anthropogenic factors contribute to the proliferation of temporal breeding sites.

**Tanghin **was formerly a rural village which has been gradually, but inadequately, urbanized since 1980. It is limited in the north by some rural villages and separated from the centre district (Dapoya) by the dam. In addition numerous spring-borne pools can be found throughout the district offering suitable habitats for mosquito development. As a relatively recent urban district, it shares some characteristics with the rural villages, such as dispersed housing pattern and the existence of spring as a water resource.

**Yamtenga **is a recent eastern peripheral non-urbanized district without formal limit with the urban districts and still slowly undergoing its urbanization process. It is characterized by condensed precarious houses built with local materials, such as mud. One permanent and large polluted-swamp occurs in the centre of the quarter constituting the principal breeding site all year long. In addition, some domestic pool or spring-borne pools are disseminated through the agglomeration constituting important breeding sites for mosquitoes.

**Zongo **is a recent peripheral non-urbanized area connecting to the town on its western side. It is characterized by dispersed houses. One major feature of this area is the presence of a natural and accidental ravine that has been exploited to collect rain water for brick making. Some small pools were disseminated in the bed of the ravine during the dry season and kept non-polluted water exposed to the sun. These water sources constituted the main breeding sites permitting the development of anophelines.

### Meteorological data

The meteorological data were provided by the National Meteorology Office located in Ouagadougou i.e. mean values of temperatures, rainfall and relative humidity for each month during the year 2006.

### Mosquito adult collections

In each study site, both traditional and modern houses were sampled for mosquito collection. During four consecutive nights, CDC traps were used to catch adult's mosquitoes in each study site between 8:00 pm and 6:00 am according to four traps inside and four others just outside of four houses randomly selected. Indoor resting females were caught by spraying four houses in proximity of the CDC-sampling houses with insecticide aerosols between 6:00 am and 9:00 am, during four consecutive days in each study site. Female mosquitoes were knocked-down onto, and immediately retrieved from, white sheets laid down on the floor of sprayed huts.

### Larva sampling and breeding sites productivity

In each study area, all water collections were numbered and prospected for the presence of anophelines larvae. A productivity index was assigned to each breeding site according to the presence or absence of anopheline larvae using the classical deeping technique [7]. As the objective was not to count the total number of anopheline larva per breeding site, we just sampled some from each positive breeding site and stored into 1.5 ml tube containing alcohol 70° dedicated to PCR analysis for species identification. When anophelines species other than *An. gambiae *s.l. were suspected, as assumed in Yamtenga large swamp and Zongo ravine, larvae were sampled and bred in an insectary until adult stage. The adults were lately emerged and identified using standard keys as described below.

### Laboratory processing of mosquitoes

Adults including both CDC and indoor spray catches and also insectary-emerged adults were identified using standard morphological identification keys [8]. When numerous specimens were collected per site, a subsample of ~30 specimens of *An. gambiae *s.l. was processed by PCR to identify species belonging the *An. gambiae *complex and molecular forms of *An*. *gambiae *s.s. [9,10]. Sporozoite rates were estimated from females of *An. gambiae *s.l. and *Anopheles funestus *collected indoors and by CDC traps, essentially in October 2006 using the ELISA CSP techniques [11].

## Results

**Figure 2:**
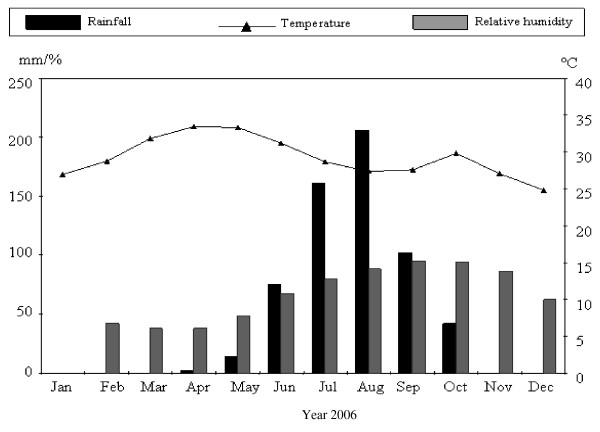
Dynamic of rainfall, relative humidity and temperatures during the year 2006 at Ouagadougou [data of National Meteorology Office, 2006]

The absolute values of the temperature exceeded 30°C in April and October. April was one of the driest months with 2.3 mm of rain and 37.7% of relative humidity while in October 41.7 mm rain with 94.3% of relative humidity were recorded (Figure 2).

### Prevalence of malaria

Except in Zongo district, malaria prevalence differed significantly between dry and rainy seasons irrespective the district (*P *< 0.05). Indeed, it was similar in Zongo for the two seasons but varied significantly between districts in dry as well as in rainy season (*P *< 0.01) and ranged from 34% to 9% (Table 1).

**Table 1:** Malaria prevalence (%) reported from the four study districts of Ouagadougou in 2004

Districts	Dry season	Rainy season	Mean
Yamtenga	9.0 [5.8-12.2]	24.6 [19.5-29.8]	16.4 [13.4-19.5]
Zongo	30.4 [24.2-36.5]	32.6 [27.2-38.1]	31.7 [27.6-35.7]
Dapoya	18.6 [11.6-25.7]	10.4 [6.6-14.2]	13.0 [9.6-16.5]
Tanghin	21.5 [16.0-27.0]	34.0 [28.1-39.9]	28.2 [24.1-32.3]

### Productivity of breeding sites

**Figure 3:**
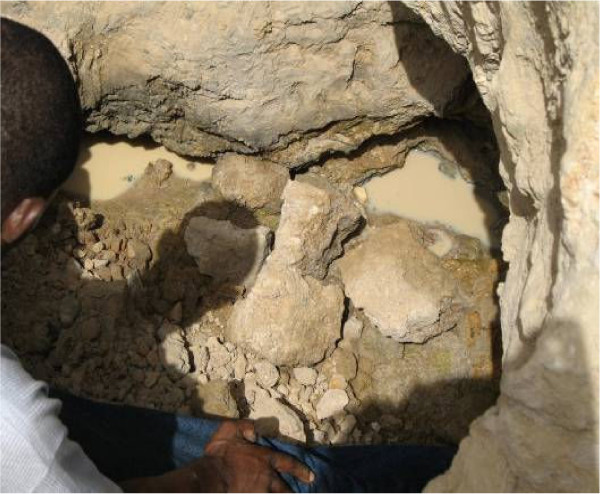
Brick-hole in Zongo district

**Figure 4:**
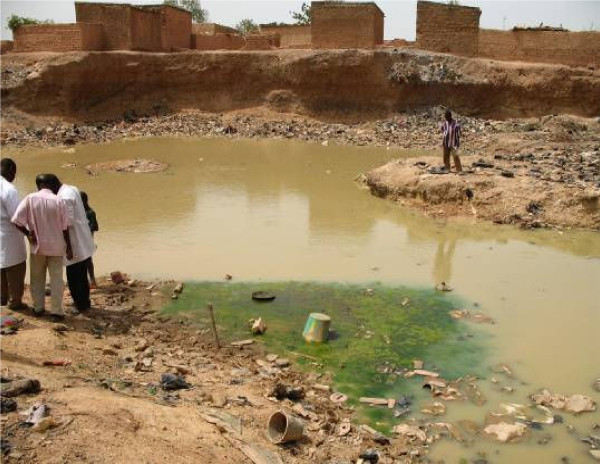
Polluted breeding site in Yamtenga district

In April 2006, no anopheline larvae were found in any of breeding sites prospected in the central district of Dapoya, both in vegetable growing wells and domestic channels, where only *Culex *sp. larvae were collected. In Tanghin district, anopheline larvae were collected only in the brick holes closer to the dam surrounding the district. Anopheline larvae were mainly collected from the breeding sites located at Yamtenga and Zongo, the two peripheral non-urbanized areas. In these two areas, 87.5% (7/8) of prospected breeding sites were found positive to anophelines. All larvae identified at adult stage as *An. gambiae *s.l. were exclusively collected from the brick-made ravine of Zongo (Figure 3) and the large polluted swamp of Yamtenga (Figure 4). Additionally to *An. gambiae *s.l., *Anopheles pharoensis *and *Anopheles rufipes *were identified among those emerged from Yamtenga swamp. In October 2006, out of 50 breeding sites observed throughout the four sites, 42 were positive for anophelines. They mostly consisted of the sites mentioned above and also additional swamps and domestic channels produced by the rainfall (Table 2).

**Table 2:** Vector breeding sites diversity and distribution throughout the study sites in 2006

Type of breeding sites						
**Districts**	***Brick hole***	***Dam***	***Channel***	***Ravine***	***Permanent swamp***	**Temporal breeding**

April 2006						

Dapoya		+	+			
Zongo	+			+		
Tanghin		+				
Yamtenga			+		+	
Total	1	2	2	1	2	8

October 2006						

Dapoya		+++	+++	+		+++
Zongo	+++		+++	+++		++++
Tanghin	++	+++	++			+++
Yamtenga	++++		++++	++	++	++++++
Total	9	6	12	6	2	16

### Anopheline density

In April 2006, except Yamtenga where two anophelines (one male and one female) were collected indoor, all mosquitoes collected both by CDC-traps and indoor sprays were exclusively composed by mosquitoes belonging to the genus *Culex *sp in all districts. In October 2006, the CDC-light traps yielded 49 anopheline mosquitoes with respectively 15, 10, 22 and 2 in Zongo, Dapoya, Tanghin and Yamtenga. Overall 110 anophelines were collected by indoor sprays vs 3,402 *Culex *composed by 95.9% *Cx. quinquefasciatus *and less than 4% *Cx. decens *(Table 3). Considering the two sampling techniques overall 159 anophelines were collected and mainly dominated by *An. gambiae *s.l. with few specimens of other species such as *An. funestus*, *An. pharoensis *and *An. rufipes*. Some sandflies identified as *Phlebotomus dubosqi *and *Sergentomya *sp. belonging to the family of *Psychodidae *were collected both by CDC and indoor sprays in all districts.

**Table 3:** CDC traps and indoor insecticide spray collections (N values)

	April 2006								
		
	Zongo		Dapoya		Tanghin		Yamtenga		
		
Mosquito genus	CDC	Indoor	CDC	Indoor	CDC	Indoor	CDC	Indoor	N value
*Anopheles *sp.	0	0	0	0	0	0	0	2	2
*Culex *sp	14	32	217	32	16	40	253	19	623
Others	3	14	0	1	6	3	14	0	41
Total	17	46	217	33	22	43	267	21	666

	**October 2006**								

	**Zongo**		**Dapoya**		**Tanghin**		**Yamtenga**		
		
**Mosquito genus**	**CDC**	**Indoor**	**CDC**	**Indoor**	**CDC**	**Indoor**	**CDC**	**Indoor**	**N value**

*Anopheles *sp.	15	31	10	4	22	49	2	26	159
*Culex *sp	234	126	763	298	642	770	252	317	3402
Others	5	2	57	9	40	32	13	3	161
Total	254	159	830	311	704	851	267	346	3722

### Species composition

Overall 115 mosquitoes were analysed in PCR to identify the species composition and to characterize the molecular forms of *An. gambiae *s.s (Table 4). Out of 44 mosquitoes analysed in Yamtenga, 59.1% were identified as *An. gambiae *M form *vs *40.9% as *An. arabiensis *without any S molecular form. In Zongo district *An. gambiae *s.l. populations were dominated by *An. arabiensis*, representing 76.6% *vs *22.3% for *An*. *gambiae *M form with only 3% of S molecular form. In Tanghin, four mosquitoes were identified as *An. gambiae *M form. Overall in April 2006, *An. arabiensis *was the predominant species (χ^2 ^= 8.72, df = 1 *P *< 0.001) reaching 59.1% *vs *40.8% of *An. gambiae *s.s., which was mainly composed by the M form (39.1%). The S form represented only 1.7% of total anophelines.

**Table 4:** Identification of malaria vector species from the four districts

	April 2006				
**Districts**	***An. gambiae *S**	***An. gambiae *M**	***An. arabiensis***	***An. funestus***	**Total**

Dapoya	0	0	0	0	0
Zongo	2 (3)	15 (22.3)	50 (76.6)	0	67
Tanghin	0	4 (100)	0	0	4
Yamtenga	0	26 (59.1)	18 (40.9)	0	44

Total	2 (1.7)	45 (39.1)	68 (59.1)	0	115

	**October 2006**				

**Districts**	***An. gambiae *S**	***An. gambiae *M**	***An. arabiensis***	***An. funestus***	**Total**

Dapoya	3 (8.1)	16 (43.2)	17 (46.0)	1	37
Zongo	4 (7.0)	24 (40.3)	30 (52.6)	0	58
Tanghin	3 (4.2)	38 (52.1)	29 (40.8)	2 (2.8)	72
Yamtenga	3 (5)	27 (67.5)	11 (27.5)	0	41

Total	13 (6.3)	105 (50.5)	87 (41.8)	3 (1.4)	208

(% in parenthesis)

Overall 206 specimens including 120 larvae and 86 females were analysed in PCR in October 2006 to identify the species composition and to characterize the molecular forms of *An. gambiae *s.s. (Table 4). They were composed by 57.8% of *An. gambiae *s.s. (51% of M form and 6.8% of S form) *vs *42.2% of *An. arabiensis*. *An. funestus *was found in low proportion (1.4%) only in Tanghin and Dapoya districts separated by the dam. The frequency of *An. gambiae *s.s. and *An. arabiensis *did not vary significantly between districts. However the M form predominated slightly (χ^2 ^= 6.97, df = 3 *P *> 0.05). The proportion of each species between April and October 2006 as significantly different (χ^2 ^= 10.23, df = 2 *P *< 0.01).

### Sporozoite rate

Out of 89 females, including *An. gambiae *M (39) and S forms (four), *An. arabiensis *(36) and *An. funestus *(three) analysed by ELISA for the presence of the circumsporozoite protein of *P. falciparum*, six were detected positive for the CSP antigen (Table 5). They were composed by three positive from Tanghin (including 2 *An. gambiae *M form and 1 *An. arabiensis*), two from Zongo (1 *An. gambiae *M form and 1 *An. arabiensis*) and one *An. gambiae *M form from Yamtenga. The global sporozoite rate was estimated higher (6.38%). Except Dapoya where no infected *Anopheles *was detected, the sporozoite rate did not differ significantly between districts (χ^2 ^= 0.48, df = 3, *P *> 0.05).

**Table 5:** Sporozoite rate of females tested by ELISA-CSP

Districts	Mosquito species	CSP-	CSP+	Total tested	Sporozoite rate (%)
Zongo	*An. gambiae M*	10	**1**	12	
	*An. gambiae S*	1	0	1	
	*An. arabiensis*	16	**1**	17	
	*An. funestus*	0	0	0	6.7 (2/30)
Yamtenga	*An. gambiae M*	7	**1**	8	
	*An. gambiae S*	1	0	1	
	*An. arabiensis*	4	0	4	
	*An. funestus*	0	0	0	7.7 (1/13)
Tanghin	*An. gambiae M*	18	**2**	20	
	*An. gambiae S*	2	0	2	
	*An. arabiensis*	13	**1**	14	
	*An. funestus*	2	0	2	7.9 (3/38)
Dapoya	*An. gambiae M*	4	0	4	
	*An. gambiae S*	0	0	0	
	*An. arabiensis*	3	0	3	
	*An. funestus*	1	0	1	0

		**83**	**6**	**89**	**6.7 (6/89)**

## Discussion

According to the parasitological values, malaria prevalence is maintained all year long in Ouagadougou town. The parasitological values differed between the two seasons revealing that malaria transmission dynamic increased intensively during the rainy season where the density of vectors also increased accordingly. This result also showed that malaria transmission varied following the districts decreasing from the peripheral to the central ones that is in accordance with our entomological data.

Indeed, some vectors from three of the four districts surveyed were detected positive to *Plasmodium falciparum *by ELISA with a relative high value of sporozoite rate especially in the periphery. The occurrence of malaria affection during the dry period could be partially due to the persistence of malaria vectors sustaining the transmission even thought they had been found in low density. The vector species composition did not differ between the two months but their relative proportion varied: *An. arabiensis *and *An. gambiae *M were the main vectors, with a low proportion of S molecular form. Few specimens of *An*. *funestus *were reported in October in Tanghin and Dapoya the most urbanised districts probably due to the presence of dams which surface was covered by vegetation suitable to *An. funestus *development. The persistence of the S molecular form in April seemed atypical and the question is how does this form to adapt to the dry season conditions under urban pattern. Many studies had shown that *An. gambiae *S molecular form is well adapted to rainy season developing in the rainfall breeding sites and survived the dried conditions by living in aestivation but such mechanisms are not well known [12]. If any, what mechanism does sustain this adaptation as only chromosomal arrangements had been pointed out as direct adaptation to the environment [13]. In fact, larvae of S form had been collected in brick-made ravine in peripheral area which ecology is considered to be similar to humid savannah. Furthermore, *An. gambiae *M form was logically the most frequent vector found during this dry season where breeding sites were mainly dominated by permanent and semi-permanent water stamps and swamps in floodable areas favourable to its development [14]. It was followed by *An. arabiensis*, which proportion had increased significantly compared to the latest study of Rossi *et al *[5]. Here, the two vectors were found in polluted breeding sites, such as permanent swamp from Yamtenga receiving domestic wastes, from where they probably exploited the clear film of water at the edge of the swamp. Thus the survival of malaria vectors during the dry season re-launches the problematic of urban malaria pointed out over the last decade [15,16]. Even though the sample size analysed was low, the sporozoite rate remained higher and mainly supported by *An. gambiae *M form and *An. arabiensis*. These results confirmed the malaria prevalence showing that the higher values were reported in the periphery. Although these results reflected those already observed in many African cities, they were important as they updated those published by Rossi *et al*. [5] since twenty years. Globally the tendency of transmission and vector distribution patterns was very similar to that reported by Rossi *et al *[5]. It can be observed that after two decades no major modifications occurred in terms of vector composition in Ouagadougou town which kept mainly the same structure especially in the periphery (Zongo and Yamtenga). That meant that the town is extending in size but basically conserves a rural landscape structure in the periphery. Accordingly the vector composition looks like that observed in the neighbouring villages in rural setting (Dabiré unpublished. data). Indeed the recent study of Wang *et al *[4] in Ouagadougou also reported a gradient of endemicity between the urban centre and the periphery and clearly linked the malaria prevalence to the seasonal availability of water supplies. The current results confirmed the distribution of the disease prevalence and provided additional information on the vector species and the sporozoite rate that is crucial to assess the risk of malaria transmission in Ouagadougou town. Herein the infection rates are higher reminding those observed in rural settings. This situation will probably progress drastically in the future with the increasing number of precarious and peripheral quarters. The occurrence of malaria vectors in urban areas in Africa is fairly well documented and local circumscribed malaria transmission had been reported in many cities [17,18]. This situation could probably be ascribed to the failure of landscape management disseminating breeding sites, thus favouring vector-human contact. Moreover, during the last decade, gardening had expanded in the peripheral belts and also in the centre of many towns disseminating numerous breeding sites favourable to mosquito development [15,19]. All these factors are contributing to maintain anophelines beyond to rainy period. In several African towns anophelines have been observed developing in wide range of habitats largely supplied to natural water resource such as lagoon or permanent swamps in Accra, Cotonou and Lagos or including polluted water dedicated to vegetable growing in Dakar [15,20-23]. But in many cases the entomological inoculation rates were reported being low decreasing from the periphery to the centre. Nowadays many people originated of rural foci not necessary free of malaria are setting in periphery of the towns which could increase the picture of "urban" malaria transmission as being observed in Ouagadougou town. This landscape modification will probably modify the pattern of malaria transmission, while control measures are, so far, focused preferentially towards rural populations. This landscape modification must be monitored and controlled by a system of planning to prevent vector proliferation.

## Competing interests

The authors declare that they have no competing interests.

## Authors' contributions

FF and DKR participated to the study design, undertook the field study, analysed data and wrote the paper. OA, CM, MPE and TKH participated to the field study and the laboratory analysis. GLC participated to the manuscript drafting. All authors have read and approved the final manuscript.
